# Mummies

**Published:** 1891-06

**Authors:** Frank G. Carpenter


### Mummies.

The great age of mummy-making in Egypt lasted about 2,700 years, and it is estimated that there are buried in Egypt more than 400,000,000 mummies, or about eighty times the present population of the Nile Valley. It took a long time to make a mummy, and the most approved methods required about 75 days. In the first place, it took 16 days to clean the body. Then 20 days more for salting and bitumenizing it. It required from 30 to 35 days for the splicing and bandaging, and the bandages were all made of linen. This linen was in strips from three to four inches wide, and it took at least a thousand yards to clothe the mummy. Every toe and every finger had to be wrapped up separately, and it took an experienced man to do the work. The mummy business of Egypt was thus on a large scale and there were great mummy factories, some of which had from 500 to 600 corpses on hand at a time. They had a way of marking each corpse with indelible ink, so that there was no danger of mixing them up, and the institutions were run on business principles. Each had a number of painters and gilders connected with it, and the work was divided among a number of different classes each of which had a specialty. The bandages were put on with gum, and the making of the coffins to contain the mummies was quite a business. It was in fact so expensive to have a good coffin that only the richest could afford it, and the poorer were buried without coffins. The paupers of the land, were in many cases, merely covered with a mineral pitch, and in others their bodies were dried and salted instead of being embalmed. Artificial eyes were put into many of the mummies, and silver gloves or finger stalls were put on their fingers to keep the nails in place. After the intestines were taken out of the body the interior was cleansed with palm wine, and it was then filled with spices.

In the above estimate of the number of mummies in Egypt only the years between 2000 B. C. and 700 A. D. are taken. The fact is that there were mummies made as far back as 3800 B. C. and there is an estimate by one antiquarian that the number of mummies in Egypt is 731,000,000. Taking the mummified animals, the number would be still larger, for you find lots of mummy dogs and mummy cats in Egypt, and a boatload or so of mummy cats were lately carried to England from Alexandria to be used as fertilizing material. FRANK G. CARPENTER.

